# Ecological and Socio-Economic Determinants of Livestock Animal Leptospirosis in the Russian Arctic

**DOI:** 10.3389/fvets.2021.658675

**Published:** 2021-04-12

**Authors:** Olga I. Zakharova, Fedor I. Korennoy, Ivan V. Iashin, Nadezhda N. Toropova, Andrey E. Gogin, Denis V. Kolbasov, Galina V. Surkova, Svetlana M. Malkhazova, Andrei A. Blokhin

**Affiliations:** ^1^Federal Research Center for Virology and Microbiology, Nizhny Novgorod Research Veterinary Institute-Branch of Federal Research Center for Virology and Microbiology, Nizhny Novgorod, Russia; ^2^Federal Center for Animal Health (FGBI ARRIAH), Vladimir, Russia; ^3^Federal Research Center for Virology and Microbiology, Pokrov, Russia; ^4^Faculty of Geography, Lomonosov Moscow State University, Moscow, Russia

**Keywords:** Arctic, climate change, forest-based classification and regression algorithm, G-rate, leptospirosis, livestock, Russia, ArcGIS

## Abstract

Leptospirosis is a re-emerging zoonotic infectious disease caused by pathogenic bacteria of the genus *Leptospira*. Regional differences in the disease manifestation and the role of ecological factors, specifically in regions with a subarctic and arctic climate, remain poorly understood. We here explored environmental and socio-economic features associated with leptospirosis cases in livestock animals in the Russian Arctic during 2000–2019. Spatial analysis suggested that the locations of the majority of 808 cases were in “boreal” or “polar” climate regions, with “cropland,” “forest,” “shrubland,” or “settlements” land-cover type, with a predominance of “Polar Moist Cropland on Plain” ecosystem. The cases demonstrated seasonality, with peaks in March, June, and August, corresponding to the livestock pasturing practices. We applied the Forest-based Classification and Regression algorithm to explore the relationships between the cumulative leptospirosis incidence per unit area by municipal districts (G-rate) and a number of socio-economic, landscape, and climatic factors. The model demonstrated satisfactory performance in explaining the observed disease distribution (*R*^2^ = 0.82, *p* < 0.01), with human population density, livestock units density, the proportion of crop area, and budgetary investments into agriculture per unit area being the most influential socio-economic variables. Climatic factors demonstrated a significantly weaker influence, with nearly similar contributions of mean yearly precipitation and air temperature and number of days with above-zero temperatures. Using a projected climate by 2100 according to the RCP8.5 scenario, we predict a climate-related rise of expected disease incidence across most of the study area, with an up to 4.4-fold increase in the G-rate. These results demonstrated the predominant influence of the population and agricultural production factors on the observed increase in leptospirosis cases in livestock animals in the Russian Arctic. These findings may contribute to improvement in the regional system of anti-leptospirosis measures and may be used for further studies of livestock leptospirosis epidemiology at a finer scale.

## Introduction

Animal leptospirosis is a re-emerging focal infectious disease (zoonosis) that is common in humans and animals globally (1–5). Over the past decades, inadequate attention has been directed toward the study of the disease and its impact on public health (6), particularly in countries with a temperate climate (7, 8). However, in recent years many reports and reviews of health organizations worldwide have highlighted leptospirosis as a growing problem as evidenced by the markedly increasing rates of mortality and incidence in both humans and animals in all continents (9, 10).

Apart from acute and chronic forms (11), genital leptospirosis is considered a specific syndrome unrelated to a systemic leptospirosis disease (12, 13) and caused by weakened leptospires that colonize urogenital organs. The transmission of pathogenic leptospires from animals to humans, and among animals within a population is influenced by numerous factors, including environmental (landscape and climatic) (14, 15) and anthropogenic (socio-economic) factors (14, 16, 17). According to some studies (18–20), climatic factors rank first among the common causes of endemicity and persistence of leptospirosis in tropical and subtropical countries (5, 21, 22). Globally, the prevalence of the disease varies from region to region depending on the geographic location. Regions and countries with high endemicity are characterized by hot humid weather, and tropical and subtropical climates, which contribute to the survival of pathogenic *Leptospira* in the external environment (23–25). In addition to the tropical climatic zones where environmental conditions are most favorable for survival of pathogenic *Leptospira*, the disease is also quite widespread among livestock in the temperate latitudes of the Eurasian continent (7, 8, 10). In the temperate zone, climatic changes (i.e., warming) could be one of the factors increasing the ability of *Leptospira* to survive in the environment (10, 25–27). Other factors contributing to the spread of infection among both humans and animals in these temperate zones are socio-economic phenomena, such as urbanization (17, 28), agricultural intensification (17, 29), as well as changes in the economic status of people, including poverty, homelessness, and even the presence of individual communities in poorly or sparsely populated urban areas (30), which may result in poor hygiene and rodent-borne infections (31–33).

Humans and animals carrying leptospires are direct sources of infection. The factors mediating transmission among livestock and humans, as well as the natural reservoirs of pathogenic *Leptospira*, are wild animals, including rodents, and the environment itself (34–36).

Scientific literature suggests that the etiological structure of the livestock with leptospirosis in Russia had not changed significantly over the last 40 years with the following prevailing serovars (37):

- In cattle: *Hebdomadis*- 34.13%, *Sejroe*- 27.25%, *Tarassovi*- 10.96%, *Pomona*- 6.65%, and *Grippotyphosa*- 6.03%;
- In pigs: *Pomona*- 41.60%, *Icterohaemorrhagiae*- 31.58%, and *Tarassovi*−14.44%;
- In small ruminants: *Icterohaemorrhagiae*- 35.62%, *Pomona*−17.75%, *Grippotyphosa*−14.84%, *Sejroe*- 7.48%, *Hebdomadis*- 6.68%, and *Tarassovi*- 6.31%;
- In equine: *Icterohaemorrhagiae*- 27.07%, *Grippotyphosa*- 22.67%, *Pomona*- 10.65%, *Tarassovi*- 10.12%, and *Canicola*−9.69%;
- In dogs: *Canicola*- 51.07% and *Icterohaemorrhagiae*- 26.86%.

This study aimed to gain a better understanding of the epidemiology of leptospirosis, particularly in the Arctic where there is a less dense livestock population and severe climate. Leptospirosis emergence under these conditions has been understudied. In this study, we analyzed the relationships between the cumulative leptospirosis incidence in livestock animals per unit area of the Russian Arctic and a number of potentially influential socio-economic and climatic factors. We also assessed the Forest-based Classification and Regression tool for predicting possible areas with an increased risk of an epidemic.

## Materials and Methods

### Study Area

**Figure 1:**
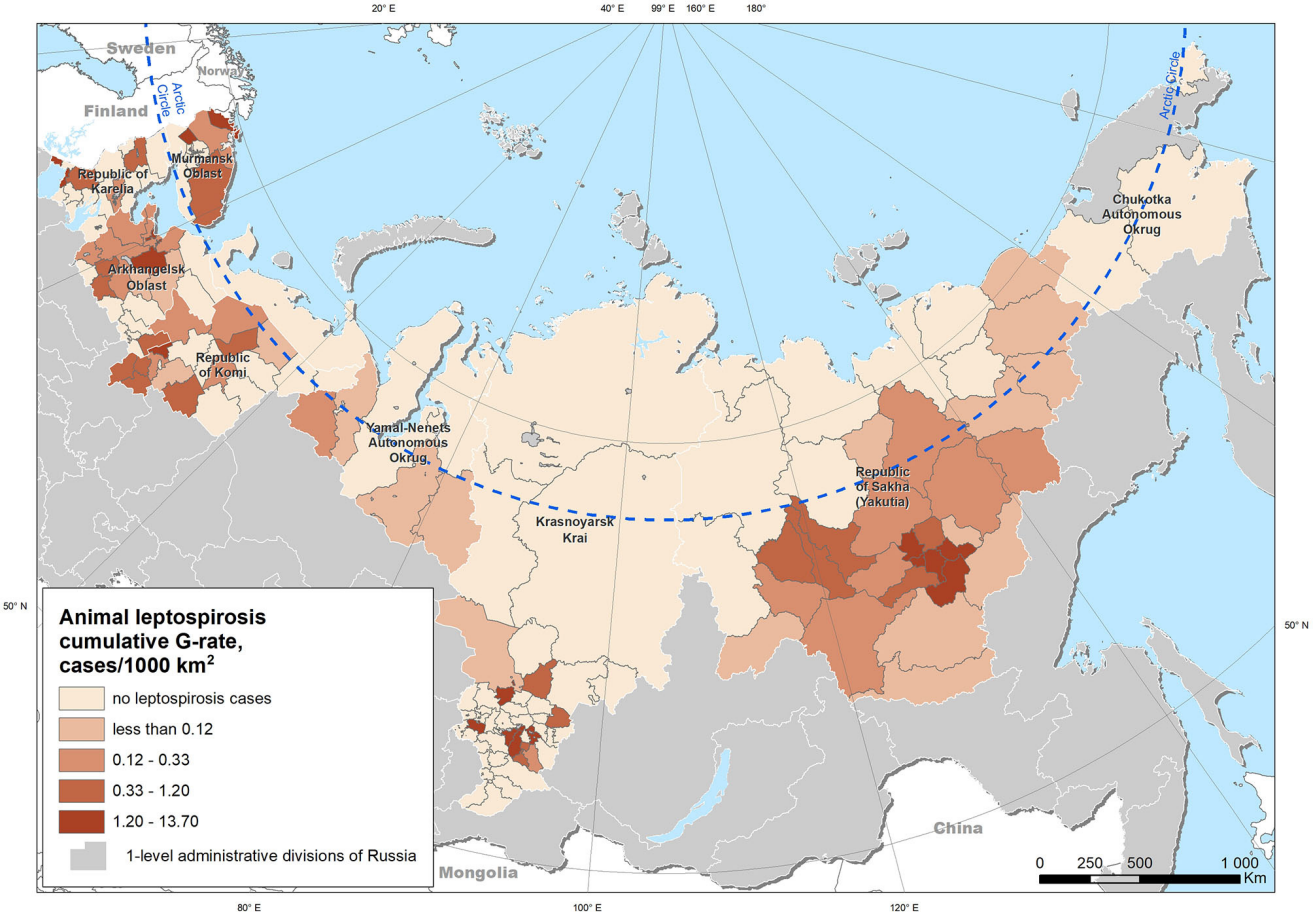
Study area and cumulative leptospirosis G-rate in livestock (cases per 1,000 km^2^) for the period from 2000 to 2019.

We studied the manifestation of leptospirosis in livestock animals in the regions located in the Arctic zone of the Russian Federation. The Russian Federation is administratively divided into 85 regions that are subdivided into secondary administrative units, which are the municipal and urban districts (hereinafter termed “districts”). The zone under consideration includes nine regions whose territories are crossed by the Arctic Circle: Arkhangelsk Oblast, Murmansk Oblast, Republic of Karelia, Republic of Komi, Yamalo-Nenets Autonomous Okrug, Nenets Autonomous Okrug, Krasnoyarsk Krai, Republic of Sakha (Yakutia), and Chukotka Autonomous Okrug. These regions are subdivided into 199 districts with areas varying from 4 to 798,000 km^2^, while the population density varies from 0 to 2,896 people per km^2^ (https://rosstat.gov.ru/free_doc/new_site/region_stat/arc_zona.html). Due to the high heterogeneity of population density, we excluded those areas with a population density exceeding the mean plus three standard deviations (i.e., representing outliers in population density) from further analysis. Such areas are represented by small but densely populated urban territories with a scarcity of agricultural livestock. Additionally, all the other districts with no livestock population were excluded. Thus, the total number of territorial units suitable for the analysis was 166. The majority of the study area lies north of 60°N; however, some of the Krasnoyarsk Krai districts extend southward, up to 52°N (Figure 1).

### Leptospirosis Data

**Figure 2:**
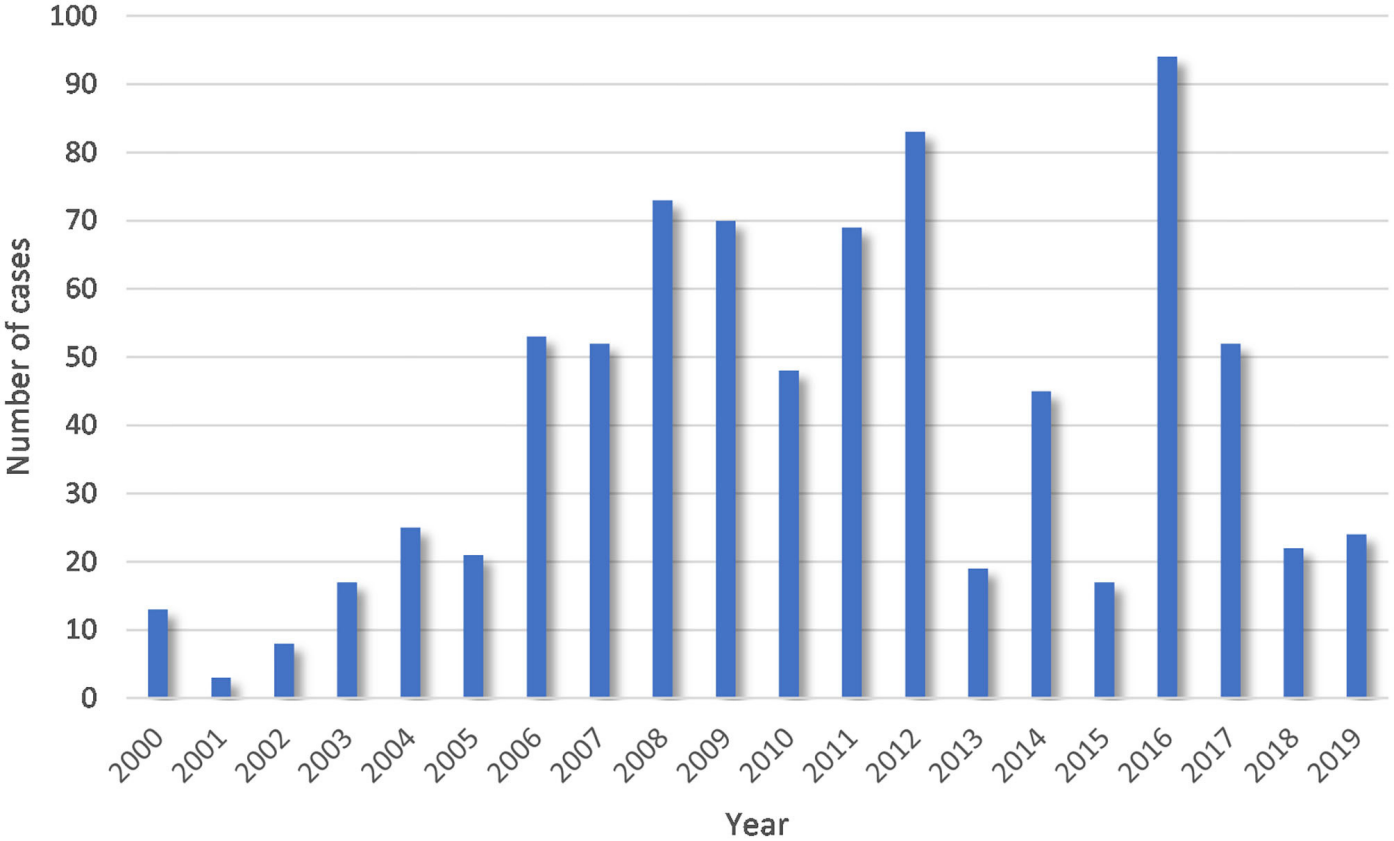
Yearly distribution of livestock leptospirosis cases for the period from 2000 to 2019.

Data on the livestock leptospirosis cases for the 2000 to 2019 period were obtained from the regional veterinary services. Herein, a case is defined as a registered, laboratory-confirmed detection of leptospirosis in a geo-referenced population of animals (a herd or a farm). Cases were detected both by herd owners and during routine government monitoring. Under the state standard GOST 25386-91 (http://docs.cntd.ru/document/gost-25386-91), laboratory confirmation was performed using the microscopic agglutination test (MAT) method with a set of 7 reference cultures: *Pomona, Tarassovi, Canicola, Hebdomadis, Sejroe, Grippotyphosa*, and *Icterohaemorrhagiae* with preliminary testing on a reference culture previously identified as typical for the specific region. The reaction was evaluated with a positive cut-off using a serum dilution of 1:50 for unvaccinated animals and 1:100 for vaccinated animals with dark-field microscopy. For previously vaccinated animals, testing was conducted at least 3 months following the vaccination. In the case of a positive MAT result with no clinical signs, confirmatory testing using real-time polymerase chain reaction was conducted (38). After excluding data with inaccurate information or a lack of geographic coordinates, the database counted 808 cases among livestock animals. Most of them were cattle (398, 49%) and horses (314, 39%). Figure 2 shows the distribution of cases by years. Leptospirosis location data were converted into a shape-file format for further modeling and processing.

### Environmental and Socio-Economic Determinants

Several socio-economic, landscape, and climatic factors acting as geospatial explanatory variables were considered as potential determinants associated with the incidence of leptospirosis in the livestock according to an extensive literature search. The factors were as follows:

- Socio-economic factors:
  1. *LSU_dens—*Livestock Unit Density index (units per 1 km^2^), calculated according to the European Union methodology [https://ec.europa.eu/eurostat/statistics-explained/index.php?title=Glossary:Livestock_unit_(LSU)]
  2. *Pop_dens—*population density, people per km^2^
  3. *Rural_prop—*the proportion of the rural population
  4. *Crop_prop—*the proportion of crop area in the total area of the region, as a factor presumably associated with the number of synanthropic rodents that are carriers of pathogenic *Leptospira*
  5. The volume of budgetary investments into the development of agriculture as a proxy for the level of financing of agriculture; relative indicators were considered: per unit area of the district (*Inv_area*) and per capita (*Inv_pop*).
- Landscape factors:
  1. *Alt—*altitude, m
  2. *Soil_pH—*soil pH
  3. *Water_prop—*the proportion of water bodies in the total area of the region
  4. *Swamp_prop—*the proportion of swamps in the total area of the region.
- Climatic factors:
  1. *Tasgod—*mean yearly air temperature, °C
  2. *Tasamplit—*mean yearly amplitude of daily air temperature, °C
  3. *Ndaytg0—*mean yearly number of days with the air temperature above 0°C, days
  4. *Prgod—*yearly precipitation, mm
  5. *Pr_tg0—*yearly precipitation for the period with the air temperature above 0°C, mm
  6. *Pr_tl0—*yearly precipitation for the period with the air temperature below 0°C, mm.

The Federal State Statistics Service Rosstat (https://eng.gks.ru/) was used as a data source for socio-economic indicators. The altitude was calculated based on the GTOPO30 digital elevation model with a spatial resolution of 30” (https://earthexplorer.usgs.gov/). The soil pH data at zero depth were taken from the ISRIC World Soil Information digital database with a spatial resolution of 250 m (39). The water bodies and swamp areas were calculated using a land-cover dataset based on the Proba-V satellite system data with an initial spatial resolution of 100 × 100 m for the 2000 to 2018 period (40).

The climatic indicators were calculated based on the data of long-term observations at meteorological stations in the Russian Federation (41, 42) for the 1981 to 2015 (“current climate”) period. Point data were interpolated and rasterized using the Kriging tool with a spatial resolution of 1 km^2^ in ArcGIS software environment. To assess the possible change in the epidemiological situation for leptospirosis due to the expected climate change for the 2081 to 2100 period, a predictive set of the same parameters was also calculated based on 14 climate models included in the international CMIP5 project (43). The climate change scenario RCP8.5 was used, which represents the “most severe” projecting, with climate forcing due to both natural processes and anthropogenic impacts (44).

Additionally, we assessed the relationship of the leptospirosis cases to a particular ecosystem using the World Terrestrial Ecosystems map, which represents a dataset with a spatial resolution of 250 × 250 m consisting of 431 classes based on the unique combinations of temperature, precipitation, landforms, and vegetation/land-cover layers (45). A circular buffer with a radius of 2.5 km was created around the location of each leptospirosis case to account for potential inaccuracy of geolocation based on the veterinary services information that provided the data. The prevailing categories of the ecosystems within the buffer zone were calculated using zonal statistics (ArcGIS, Esri).

### Assessment of the Relationship Between Leptospirosis Incidence and Geospatial Factors

The cumulative number of leptospirosis cases for the entire observation period per unit area by the districts (G-rate) was chosen as a measure for the intensity of the leptospirosis epidemic within the study area (46–49). The Forest-based Classification and Regression method was applied to identify the relationships between the log-transformed G-rate and a set of potential explanatory factors (50, 51). This method is a supervised machine learning approach that uses a set of decision trees built using the observed values and variables in order to create a classification (in case of categorical variables) or a regression (for numeral variables). The method is based on the construction of a large number of decision trees, each resulting from a sample obtained from the initial training sample using bootstrapping (52). The final model was selected based on a majority vote. The regression estimation was performed by averaging the regression scores of all the individual trees. The advantage of this method is the ability to work with both continuous and categorical variables (in our study, only continuous variables were used), as well as elimination of overfitting of the model. In the present study, we used 1,000 decision trees for model training, with four randomly sampled variables per decision tree, and 1,000 decision trees for validation, with 25% of the input data randomly selected for validation. The initial training and validation of the model were performed for areas with non-zero cases of leptospirosis and with non-zero livestock units number (*N* = 71). The quality of the regression model was assessed using the coefficient of determination (*R*^2^), which shows the proportion of data variation explained by the model. The *R*^2^ was reported for: (1) training data, (2) validation data, and (3) the overall model prediction for the training districts. The model returns explanatory variables' importance metrics providing the “importance” and “percent” values. The former is based on the sum of all Gini coefficients, which could be assumed as the number of times a variable is responsible for a split, and the impact of that split divided by the number of decision trees, while the latter represents as the percentage of a given variable's Gini coefficients of the total sum of Gini coefficients (53, 54).

The absence of spatial clustering of regression residuals was verified using Global Moran's I index. The values of this index, which are near zero at a high *p*-value, confirm the null hypothesis of a random spatial distribution of the residuals. Furthermore, the model was used to predict the values in the rest of the study area, both using the parameters of the current climate and the projected climate. To provide accuracy of predictions, only those districts of the study area with the values of the primary explanatory factors within the range defined by the training districts (*N* = 151) were used. To visualize the expected change in the G-rate under a future climate, a map was created, which showed the ratio of its predicted to its current value for the training and predictive regression model.

### Assessment of the Seasonality of Leptospirosis Emergence

To determine the seasonality of leptospirosis emergence, the seasonality index S was used and was calculated as the number of cases for a given month averaged over several years divided by the average annual number of cases for the corresponding year (55, 56). Additionally, seasonality was visualized using a radar chart.

### Statistical Analysis

The statistical analysis of data was carried out using MS Office Excel (Microsoft, Redmond, WA, USA) with the @Risk v 4.5 simulation analysis package (Palisade Inc., Ithaca, NY, USA). The Forest-based Classification and Regression analysis as well as other spatial data processing and visualization were performed using the geographic information systems ArcGIS Pro 2.6.1 and ArcMap 10.8.1 (Esri, Redlands, CA, USA).

## Results

### Epidemiological Analysis

Between 2000 and 2019, 808 cases of leptospirosis in livestock were recorded in the subarctic and Arctic regions of Russia. Based on the distribution of the detected cases by year (Figure 2), there appears to be three periods during which an increase in incidence was observed: 2000 to 2005, 2006 to 2013, and 2014 to 2019.

The calculation of the seasonality index revealed the prevalence of the relative number of cases in March as S = 1.53 (95% CI: 0–4.00), in June as S = 2.27 (0–5.76), and in August as S = 1.73 (0–9.6).

### Spatial Analysis and Regression Modeling

**Figure 3:**
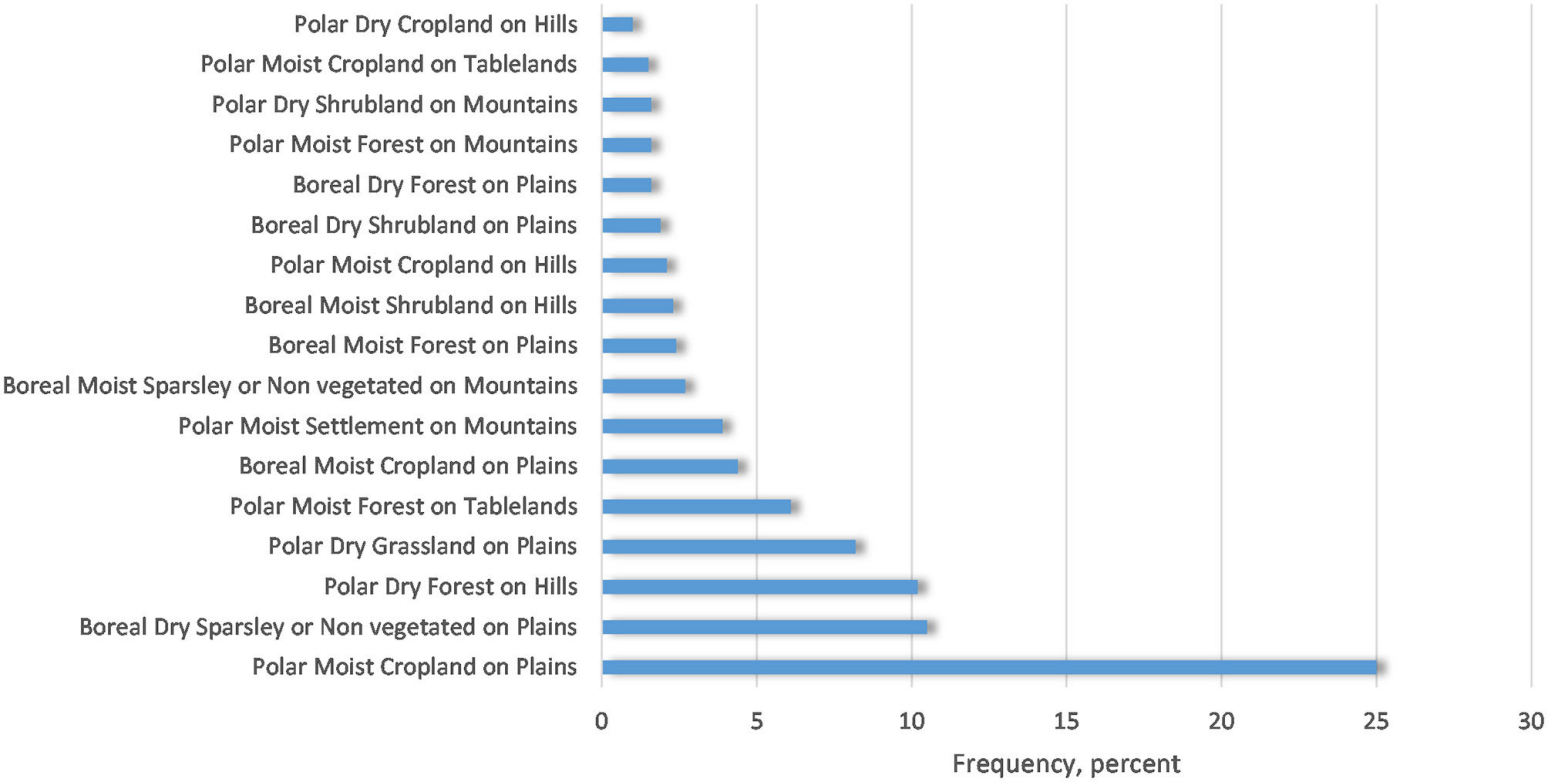
Distribution of the leptospirosis cases in relation to ecosystems.

An analysis of location of the leptospirosis cases, considering a 2.5-km buffer zone reflecting the possible uncertainty in geolocation, showed that all cases occurred in the polar or boreal climatic regions, with a predominance of croplands, forests, shrublands, and settlements as land-cover types. Most of the cases occurred in the “Polar Moist Cropland on Plains” ecological zone (Figure 3). Thus, even the most southern of the analyzed cases may still be considered as having an Arctic climate.

**Figure 4:**
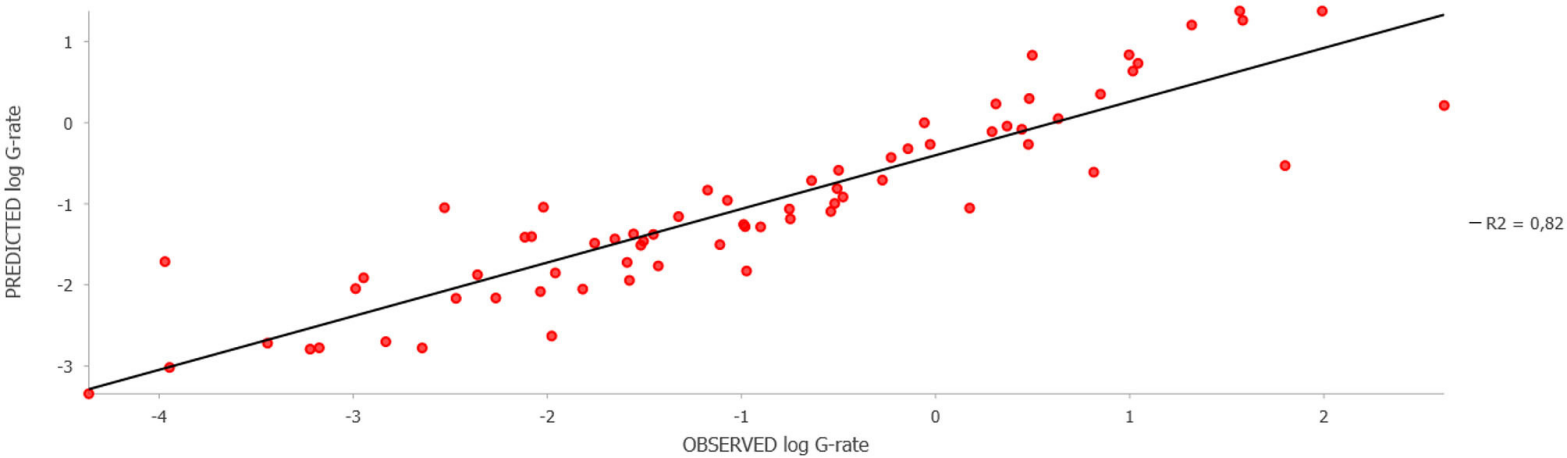
Observed vs. Predicted animal leptospirosis log G-rate as per the model fit to the training districts.

Training of a model based on the Forest-based Classification and Regression algorithm using known data on the leptospirosis incidence (G-rate) showed a high model fit to the training data (*R*^2^ = 0.94 ± 0.03; *p*-value ≤ 0.001) and an acceptable fit to the validation data (*R*^2^ = 0.53 ± 0.10; *p*-value ≤ 0.001). The relative importance of the variables based on the simulation results is shown in Table 1. The results clearly demonstrated that socio-economic factors (population density, the proportion of cropland, the density of agricultural livestock, and financial investments in agriculture per unit area) were of the greatest importance for explaining the observed distribution of leptospirosis cases, while the role of climatic and landscape factors was less significant. The test of regression residuals for spatial autocorrelation showed no tendency for residual clustering (Moran's I = −0.003; z-score = −0.227; *p*-value = 0.820). Comparison of the predicted and observed log G-rate demonstrates a satisfactory model fit to the training data with *R*^2^ = 0.82, *p*-value < 0.05 (Figure 4).

**Table 1:** Relative importance of variables based on random forest-based classification and regression analysis results.

**Variable**	**Importance**	**%**
Population density	24.3	21
The proportion of crop area in the total area of the region	15.71	14
Livestock Unit Density index	14.55	13
Budgetary investments into the development of agriculture per unit area	12.99	11
Yearly precipitation for the period with the air temperature above 0°C	5.04	4
Mean yearly air temperature	4.97	4
Yearly precipitation	4.52	4
Mean yearly number of days with the air temperature above 0°C	4.45	4
Proportion of the rural population	4.13	4
Altitude	4.08	4
Mean yearly amplitude of daily air temperature	4.04	4
Proportion of swamps in the total area of the region	4.01	4
Soil PH	3.89	3
Proportion of water bodies in the total area of the region	3.87	3
Yearly precipitation for the period with the air temperature below 0°C	2.7	2

**Figure 5:**
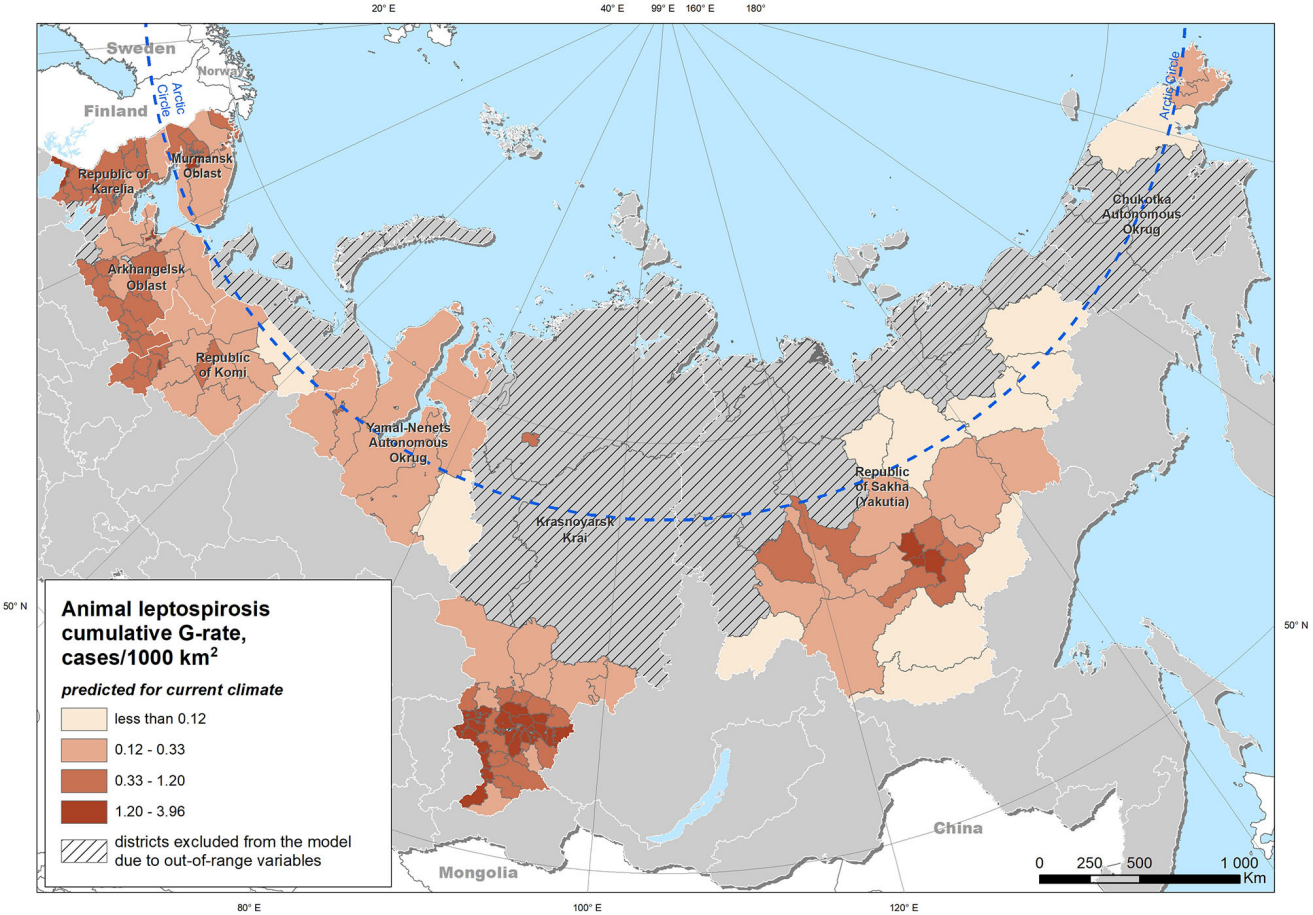
Distribution of the predicted density of leptospirosis cases (G-rate) under the current climate conditions.

**Figure 6:**
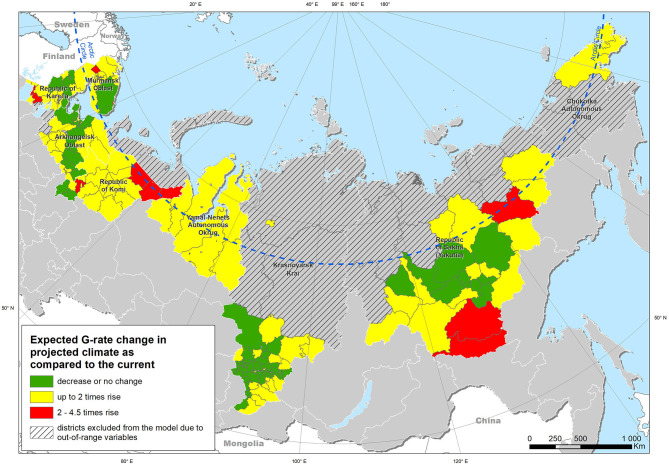
Expected change in the density of leptospirosis cases under projected climate conditions in comparison with the current climate, based on modeled changes in climatic factors.

Extrapolation of the model obtained from the entire study area under the “current” climatic conditions yielded a map of the expected case density (Figure 5). Modeling using climatic indicators for the projected climate for the period up to 2100 demonstrated an expected increase in the risk of leptospirosis in most of the study area (Figure 6). The greatest increase in the leptospirosis risk was observed in the northern part of European Russia and Western Siberia. In some of these areas, the climate-dependent risk of leptospirosis was predicted to increase by more than 4-fold.

## Discussion

In this study, we investigated the epidemiology of leptospirosis in livestock in the Arctic zone. We demonstrated the utility of the Forest-based Classification and Regression method as a tool for obtaining valuable information on the significance of factors contributing to the occurrence of leptospirosis, as well as their possible changes under various climate scenarios in the Arctic and subarctic regions of Russia. Despite the formal discrepancy between the southern part of the model region and the concept of the “Arctic zone,” our analysis demonstrates that all the considered cases of leptospirosis occurred in the polar and boreal climatic zones. Considering the selected scale of the study, socio-economic factors including population density of this region and the intensity of agricultural activity were associated with leptospirosis in this territory. In terms of landscape and climatic factors, the precipitation and temperature regime, including the mean yearly number of days with temperatures above 0°C, were identified as predominant climatic determinants, although they provided significantly lower contributions as compared to the above socio-economic variables. We also show the possible risk of future changes in the leptospirosis epidemic situation under the most unfavorable climate change scenario.

Our results support previous reports that agricultural intensification may increase the outbreaks of zoonotic diseases, such as leptospirosis (57–62). However, while the high density of commercial dairy farms similarly increased the risk of infection in both urban and rural areas, investment (insufficient fund infusions) in agriculture has an equally significant impact on the formation of risk zones.

Leptospirosis is characterized by seasonality to some extent, although individual cases of the disease do occur throughout the year. By some accounts (10, 25), in temperate climates, the summer seasonality of leptospirosis is expressed as disease in cattle, which is explained by animals being on pastures more often in this season leading to a wider contact with leptospirosis carriers and alimentary transmission of the pathogen through fodder and water from open reservoirs (26, 27, 63). Based on the epidemiological analysis of monthly data throughout the year, we observed a pronounced seasonality of the disease in the spring–summer period. This pattern is regularly repeated over the years due to changes in climatic conditions, faunistic, and economic-organizational factors, which leads to activation of the mechanism of pathogenic transmission from their source to susceptible animals. The seasonality analysis suggests that the incidence of leptospirosis in livestock has two peaks—it starts in January and gradually increases to March (15.13%) and then declines until May. The peak incidence in March may be associated with the active migration of synanthropic rodents from natural wintering areas, where food supplies are depleted, to human habitats and livestock keeping areas, which entails an increase in contact with livestock animals. The second peak in incidence is observed in June (18.32%), which may be associated with climatic factors favorable to the accumulation of the causative agent in the external environment and the active interaction of the susceptible livestock with leptospirosis carriers.

Based on our results, climatic factors showed a significantly lower correlation with the areas showing an increased concentration of leptospirosis cases. This could be explained, firstly, by the selected scale of the study implying that the socio-economic variables have a predominant influence on the scale of the whole region, determining the intensity of the epidemic manifestation of the disease. Nevertheless, the most significant indicator among climatic factors was the mean yearly number of days with air temperatures above 0°, which was in good agreement with the previous studies' results (63–66). The next most important climatic factors were also indicators related to air temperature: the mean yearly amplitude of daytime temperature and the mean yearly air temperature. A significant relationship was also revealed with the proportion of swamps in the total area of the region, which is consistent with previously recognized environmental factors conducive to leptospirosis (57, 63, 67).

A total number of 808 leptospirosis cases over the whole study period of 20 years yields relatively small average yearly numbers per study district (<1) that makes it difficult to implement space-time regression models. Hence, in our study we considered the aggregated number of cases per unit area throughout the whole study period, which provides response variable values more suitable for modeling.

The Forest-based Classification and Regression method is a popular machine learning method used in classification and forecasting. This tool allows analysts to easily incorporate tabular attributes, features, and explanatory rasters when building predictive models, and expands predictive modeling capabilities to be accessible to all geographic information system (GIS) users. Our research demonstrated the effectiveness of the spatial Forest-based Classification and Regression algorithm for analyzing results under various climate change scenarios. The integration of the Forest-based Classification and Regression tool as a spatial algorithm for exploring these factors makes it easier to consider different scenarios than using traditional regression methods due to reduced demands on the input data distribution and format.

Nevertheless, the study had some limitations in terms of the method used. First, there were a limited number of spatial units available for analysis. As a data-driven method, the Forest-based Classification and Regression tool performs better with large datasets [at least several hundred input analysis units according to the method guidance provided by the software producer (68), while in our study, we used 71 districts for model training and 151 districts for prediction]. However, some studies have reported successful implementation of this approach on relatively small datasets (69, 70). Increased number of trees (1,000 in our study) allows reducing out-of-bag errors, which represent portions of data not participating in trees' construction. Second, there is uncertainty regarding the binding of specific values to a territorial unit, as the variation in climatic parameters presented in raster form within the districts can be significant. The socio-economic factors, expressed as density indicators over the entire territory of the region, may also inadequately reflect the true significance of the factor in the places of actual registration of leptospirosis.

It should also be noted that our forecast is based only on the expected changes in climate in the future, which according to our model has a significantly smaller effect on the concentration of leptospirosis cases than the socio-economic determinants. Thus, a change in the structure of animal farming, expansion of cultivated areas, financial support for agriculture, and the occupation of new territories could collectively have a much stronger impact on the leptospirosis incidence outweighing the influence of climatic factors.

## Conclusions

Our study provided empirical evidence that the factors involved in transmission of leptospirosis in the Arctic and subarctic regions of Russia are complex and include environmental and socio-demographic indicators. The assessment of the significance of the main factors (socio-economic and climatic) when using the Forest-based Classification and Regression method indicated that the main contribution to the increased incidence of leptospirosis is from socio-economic conditions related to the population agricultural activity. Indicators of precipitation and temperature regime were the predominant contributors among the climatic factors.

The information obtained in this study on the risk factors for livestock leptospirosis outbreaks supports the One Health approach to animal disease prevention and control, which takes into account anthropogenic factors, animal density distribution factors, and the environment. Future research should be specifically designed to assess the impact of interventions under different scenarios.

## Data Availability Statement

The raw data supporting the conclusions of this article will be made available by the authors, without undue reservation.

## Author Contributions

OZ, AB, and FK: conceptualization. FK and OZ: methodology, software, writing—original draft preparation, and visualization. AB and FK: validation. FK, OZ, and GS: formal analysis. OZ and NT: investigation. NT, SM, and GS: resources. FK and AG: data curation. FK, AB, OZ, and SM: writing—review and editing. AB, II, and AG: supervision. AB, FK, and DK: project administration. All authors have read and agree to the published version of the manuscript.

## Conflict of Interest

The authors declare that the research was conducted in the absence of any commercial or financial relationships that could be construed as a potential conflict of interest.
